# Management of Hypoplastic or Hypomineralized Defects with Resin Infiltration at Pediatric Ages: Systematic Review

**DOI:** 10.3390/ijerph20065201

**Published:** 2023-03-15

**Authors:** María Dolores Casaña-Ruiz, Laura Marqués Martínez, Esther García Miralles

**Affiliations:** Dentistry Department, Faculty of Medicine and Health Sciences, Catholic University of Valencia San Vicente Mártir, 46001 Valencia, Spain

**Keywords:** infiltration, ICON, pediatric dentistry, minimal invasive treatment

## Abstract

Hypoplastic or hypomineralized enamel defects represent a recurrent reason for consultation within the pediatric population, causing great discomfort due to their aesthetic appearance, as well as their functional limitations. Current conservative dentistry requires minimally invasive treatments in order to treat such defects and provide successful, definitive solutions. A systematic review of the literature has been carried out in accordance with the PRISMA recommendations. A search was carried out in the PubMed, Scopus, SciELO and Web of Science databases, completed with a manual search. The following variables were extracted from the selected studies: author, year, publication journal, type of study, sample, age of the participants and the materials used for its development. From the initial electronic search of the four databases, 282 articles were identified: 34 from PubMed, 240 from Scopus, 0 from SciELO and 8 from Web of Science. After eliminating duplicate articles, a total of 225 remained. After reading the title and abstract, 158 articles were eliminated, leaving 68. Upon reading the full text, the remaining studies were eliminated for not answering the research question or the inclusion criteria, leaving a total of 13 articles. Finally, 12 articles were used to carry out the systematic review. Treatments performed to date with the ICON™ system in pediatric patients have shown good results after their application. Since the variability of diagnostic methods has been observed, new diagnostic and assessment protocols should be created after treatment to objectify their effect on hypoplastic or hypomineralized enamel defects. In the same way, it has been described that treatment provides better results if combined with other opalustre-type or remineralizing materials. This review is registered in PROSPERO with the number CRD42021288738.

## 1. Introduction

Hypoplastic or hypomineralized enamel defects are a frequent finding in the pediatric population and represent a significant challenge, both due to the aesthetic compromise, and because they favor the formation of caries, either in the primary or in the permanent dentition. Pediatric dentists must be aware of the risk factors and offer conservative treatments that reduce the visualization of such defects, thus improving their patients’ quality of life [1].

For the treatment of hypoplastic or hypomineralized enamel defects, multiple options are available, from more conservative techniques to more invasive ones. The severity of the injuries is a determining factor in selecting the appropriate option. However, its etiology will not be an excluding factor for the choice of treatment [2].

Enamel defects were first identified in 1901. Since then, numerous indices and classifications have been developed for their correct diagnosis. Due to the development of the EAPD (European Academy of Pediatric Dentistry) criteria, the nature and origin of EEDs have been diagnosed with increasing certainty, but the validation of the classification methods is needed, as well as their reliability and feasibility. Quantitative defects, known as enamel hypoplasia, occur as a result of the insufficient formation of the enamel matrix, revealing a decrease in the amount of enamel formed [1].

The local factors causing this alteration are summarized as a possible trauma, which can lead to enamel hypoplasia. Among the systemic factors can be found nutritional deficit, neonatal diseases, delay in childbirth, congenital syphilis and stress [3].

Hypoplastic defects can affect both temporary and permanent dentitions, and clinically, they can present as cracks or pits rough to the touch. 

Qualitative defects, known as hypomineralizations, consist of alterations in the opacity of the enamel without a reduction in its thickness [3].

There are different available treatments to solve such hypoplastic or hypomineralized defects, both for functional and aesthetical purposes. An alternative and promising therapy, which at first, was thought to be exclusively for the treatment of carious lesions, could be the infiltration of the defects with low-viscosity light-curing resins. The resin fills a large majority of the porous voids in enamel, creating a refractory index that is similar to sound enamel. Currently, the only product on the market that uses this approach is Icon^®^, which contains special resins, optimized for rapid capillary penetration into defective enamel [4].

Due to the simplicity of the application technique, as well as the growing interest that minimally invasive techniques have generated in recent years, its use in pediatric patients could currently be highly advantageous and recurrently used by pediatric dentists.

The objective of this study is to assess the effectiveness of infiltrated resins, compared to other methods of minimal intervention in terms of clinical effectiveness and aesthetic improvement.

## 2. Materials and Methods

A systematic review of the literature has been carried out in accordance with the PRISMA recommendations (PRISMA 2020 (Predefined Reporting Items for Systematic Reviews and Meta-Analyses; The PRISMA 2020 statement: an updated guideline for reporting systematic reviews) [5]. The data was reported following the structure and content dictated by the 27 items included in the statement.

The review protocol has been registered in PROSPERO with the number CRD42021288738.

Eligibility criteria. Eligible studies were those that treated hypoplastic or hypomineralized enamel defects in pediatric patients using infiltrated resins, ICON^®^. The inclusion criteria were studies in humans, particularly in children up to 17 years and 11 months (pediatric age) where the use of infiltrated resins was used as a treatment. 

Randomized clinical trials (RCTs), longitudinal studies, cohort or case-control studies, both retrospective and prospective, in vitro or in vivo were included. No restrictions were established regarding the year of publication or language.

The objective was to answer the following research question: Do clinical and aesthetic results (O) improve when using infiltrated resins (I) in pediatric patients with hypoplastic or hypomineralized enamel defects (P)?

In order to identify the most relevant studies, four different electronic databases were used: PubMed, Scopus, SciELO and Web of Science. In specific cases, the authors of the articles were contacted via email in order to request additional information. In addition, the references of the resulting studies were scanned for potentially eligible studies that did not appear in the preliminary database search. This review was last updated in September 2022.

The search strategy was designed considering previous studies in the field and their most cited descriptors. The keywords to identify the articles were: “pediatric* dentistr*” or “paediatr* dentistr*” or “child* or infant* or temporary or deciduous* AND ICON^®^ or infiltration*” or “dent* infiltration*” or “infiltrant*” AND “minimal* intervention” or “minimally invasive treatment”. 

References identified using this search strategy were exported from each database to Mendeley Reference Management software v 1.19.8 (Elsevier, Amsterdam, The Netherlands) to check for duplicates. After ruling out duplicates, two reviewers (MD-CR and L-MM) independently assessed the titles and abstracts of all identified articles. In case of discrepancy between them, a third author (E-GM) was consulted. If the abstract did not provide enough information to make a decision, the reviewers read the full article. Finally, those that met the requirements were incorporated into the study.

The data synthesis of the included studies was divided into variables for study characteristics, methodology and results. To identify the characteristics of the studies: author, year and journal of publication. Regarding their methodology, the type of study, sample, age of the participants and the materials used for its development were assessed. The outcome variables included: the significant results found and the conclusions drawn from each study analyzed (Table 1).

Table 2 presents the results of the included studies that produced significant differences regarding the improvement in quality of life by eliminating opacities or enamel defects of the upper anterior teeth. 

Studies included in this review were independently assessed for internal methodological risk of bias. The PEDro scale was used for experimental studies and RCTs and the SCED scale for clinical cases [6] (Table 3, Table 4 and Table 5).

**Table 1:** Study methodology of the studies included in this review.

Author. Year	Study	Methodology	Material Used	Statistical Treatment
Hasmun, N. 2020 [7]	Experimental longitudinal intervention study.	Adapted survey.	Child Oral Health Impact Profile Short Form 19 questionnaire.	(SPSS v24.0, IBM Corp., Chicago, IL, EE. UU.)
Moradi, S. 2021 [8]	Observational cross-sectional study	Original survey on dental procedures in dental and postgraduate students.	Adapted thanks to the International Caries Consensus Collaboration (ICCC)	Frequencies and percentages.
Memis B. 2015 [9]	Experimental “in vitro” study	Power analysis (Power and Precision software ver. 4, Biostat, Englewood, NJ, USA	Stereomicroscope (Leica MZ12, Leica Microsystems, Wetzlar, Germany	SPSS software (ver. 20; Chicago, IL, USA). Mann–Whitney U test. Kruskal–Wallis
Swamy, D. 2017 [10]	Experimental “in vitro” study	Extracted and treated teeth to assess penetration	Stereomicroscope (20x, Stemi SV 11, Carl Zeiss, Oberkochen, Germany)	SPSS (SPSS Inc., Released 2009. PASW Statistics for Windows, Version 18.0. Chicago, IL, USA
Bhandari, R. 2019 [11]	RCT “in vivo”	Randomized clinical trial	CIELAB method	ANOVA and post hoc Tukey’s test
Mattos-Silveira, J. 2015 [12]	RCT	Parallel groups	Wong-Baker faces scale	SPSS software (ver. 20; Chicago, IL, USA
Hasmun, N. 2018 [13]	Experimental prospective intervention study	Survey	Child Oral Health Impact Profile Short Form 19 questionnaire	(SPSS) v24.0 (IBM Corp., Chicago, IL, USA
Bagher, S.M 2018 [14]	RCT Split mouth	Test—control Temporary molars.	NaF alone in the control group or combined with resins.	NA
Turska-Szybka A 2014 [15]	Experimental “in vitro” study	Extracted primary molars shoving white spots on smooth surfaces	Vickers micro-hardness test. Depth of infiltration and microhardness were evaluated.	NA
Kabaktchieva, R. 2014 [16]	Experimental “in vivo” study		Light-induced fluorescence (SoploLife chamber)	NA
Muñoz M. 2012 [17]	Case report	NA	Dean’s classification7 system	NA
Ammari, MM 2017 [18]	RCT Split mouth	CliniView	Facial Image Scale Cariogram model	SPSS software (SPSS Inc., Chicago, USA-version 22)

NA: Not applicable.

**Table 2:** Study results of significance level.

Studies	Significance Level	Conclusions
Hasmun, N. 2020 [7]	*p* < 0.001	Quality of life is significantly improved.
Memis B. 2015 [9]	*p* ≤ 0.05	Fluoride varnish + resin infiltration significantly inhibited the progression of the lesion in deciduous teeth.
Bhandari, R. 2019 [11]	*p* ≤ 0.001	Microabrassion CPP-ACFP, bring better esthetic results.
Hasmun, N. 2018 [13]	*p* ≤ 0.05	Stain removal, positive impact on children’s well-being.
Bagher, S.M 2018 [14]	*p* = 0.04	Resin infiltration + NaF, better results.
Turska-Szybka A 2014 [15]	-	Icon^®^ infiltrates at least half the depth of enamel lesions in deciduous teeth.

**Table 3:** Observational cross-sectional study quality, STROBE statement.

**Title and Abstract**	**1**	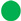
Introduction Context/basics	2	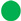
Objective	3	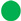
Methods Study design	4	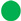
Context	5	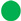
Participants	6	-
Variables	7	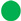
Data sources	8	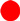
Biases	9	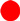
Sample size	10	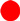
Quantitative variables	11	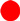
Statistical methods	12	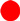
Results Participants	13	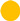
Descriptive data	14	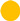
Data of the results variables	15	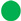
Participants results	16	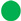
Other analysis	17	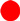
Discussion Key results	18	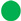
Limitations	19	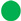
Interpretation	20	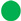
Generalizability	21	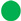
Other information Financing	22	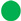
Total	-	13/22

Green circle—Appears; Orange circle—Partially appears; Red circle—Not appears.

**Table 4:** Quality of experimental studies and RCTs; PEDro scale.

Items	Hasmun, N. 2020 [7]	Bhandari, R. 2019 [11]	Hasmun, N. 2018 [13]	Bagher, S.M. 2018 [14]	Swamy, D.F. 2017 [10]	Ammari, MM 2017 [18]	Mattos-Silveira, J. 2015 [12]	Memis B. 2015 [9]	Turska- A 2014 [15]	Kabaktchieva, R. 2014 [16]
The selection criteria were specified	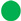 pg.7	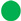 pg.2	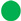 pg.3	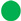 pg.4	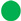 pg.1	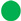 pg.2	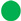 pg.2	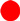 v pg.2	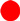 v pg.2	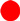 v pg.2
Subjects were randomly assigned to groups	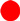 pg.7	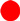	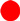 v pg.5	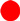 v pg.6	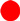 v pg.2	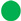 pg.6	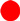	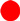 v pg.2	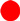 v pg.2	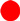 v pg.2
The allocation was hidden	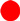 pg.7	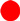	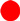 pg.5	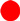 pg.6	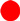 v pg.2	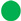 pg.6	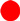	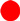 v pg.2	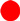 v pg.2	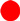 v pg.2
The groups were similar at baseline with respect to the most important prognostic indicators.	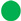 pg.7 y 10	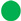 pg.4	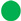 pg.4	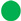 pg.5	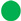 pg.2	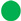 pg.3	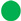 pg.3	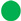 pg.2-3	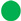 pg.2-3	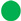 pg.3
All subjects were blinded.	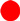 pg.8	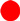	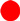 pg.5	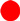 pg.6	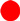 pg.2	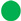 pg pg.6	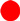	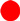 v pg.2	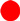 v pg.2	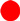 v pg.3
All therapists were blinded.	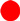 pg.8	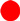	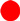 pg.5	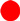 pg.6	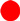 pg.2	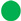 pg.2	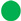 pg.2	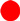 v pg.2	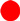 v pg.2	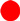 v pg.3
All assessors were blinded.	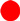 pg.8	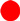	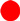 pg.5	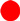 pg.6	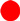 pg.2	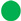 pg pg.2	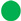 pg.2	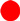 v pg.2	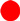 v pg.2	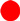 v pg.3
Means were obtained from more than 85% subjects.	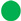 pg.10	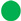 pg.4	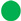 pg.6	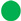 pg.7	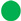 pg.3	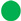 pg.4	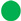 pg.4	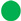 pg.4	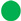 pg.4	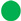 pg.3
Results from all subjects were presented.	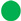 pg.10	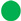 pg.4	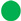 pg.6	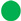 pg.7	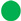 pg.3	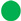 pg.4	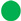 pg.4	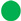 pg.4	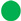 pg.4	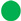 pg.3
Statistical comparison results between groups were reported for at least one key outcome.	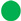 pg.9	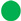 pg.4	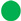 pg.7	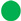 pg.8	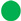 pg.2	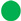 pg.2	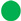 pg.3	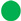 pg.4	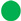 pg.4	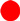 pg.3
The study provides point and variability measures for at least one key outcome	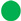 pg.9	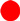	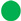 pg.7	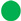 pg.8	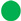 pg.3	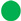 pg.2	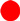	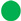 pg.4	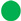 pg.4	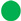 pg.3
Total:	6/11	5/11	6/11	6/11	6/11	11/11	7/11	5/11	5/11	4/11

Green circle—yes; Red circle—not.

**Table 5:** Quality of Experimental studies, SCED scale.

**1.Clinical History**	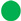
2.Target behaviors	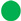
3.Desing	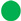
4.Baseline	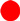
5.Treatment behavior	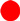
6.Raw data	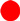
7.Interrater reliability	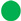
8.Independence of assessors	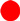
9.Statistical analysis	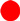
10.Replication	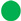
11.Generalizatino	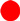
Total	5/11

Green circle—yes; Red circle—not.

## 3. Results

### 3.1. Study Selection and Flow Diagram—Study Results

**Figure 1:**
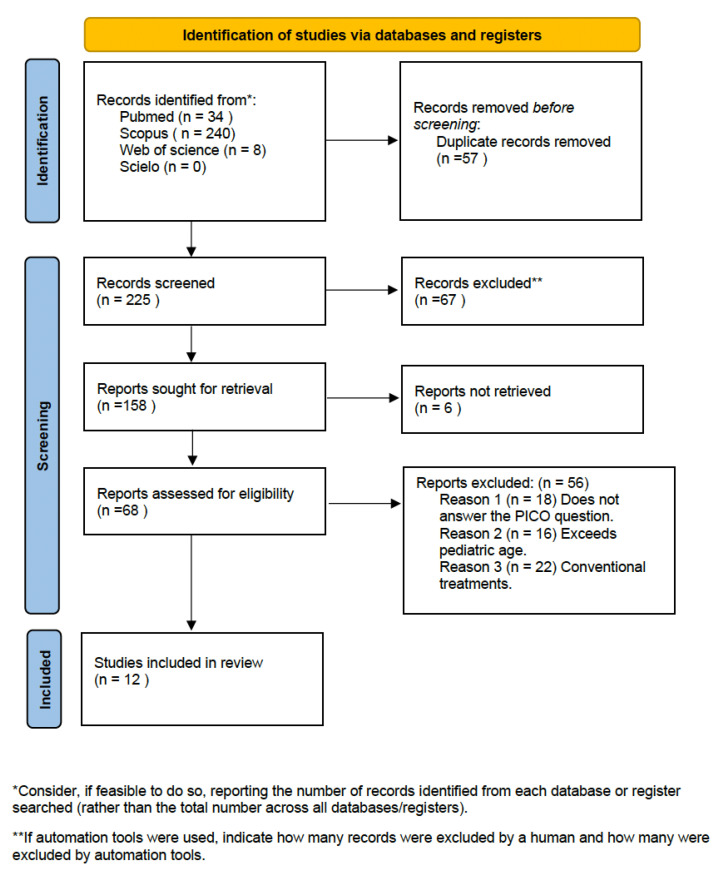
PRISMA flowchart of study selection process.

From the initial electronic search of the four databases, 282 articles were identified: 34 from PubMed, 240 from Scopus, 0 from SciELO and 8 from Web of Science. After eliminating duplicate articles, a total of 225 remained. After reading the title and abstract, 158 articles were eliminated, leaving 68. After reading the full text, others were eliminated for not responding to the research question or the inclusion criteria, leaving a total of 13 articles. Finally, 12 articles were used to carry out the systematic review. The PRISMA flowchart (Figure 1) provides an overview of the article selection process.

### 3.2. Results of Individual Studies, Meta-Analysis and Additional Analyses

For the evaluation of the influence on the improvement of quality of life, adapted surveys and their subsequent statistical treatment were used [7,8].

With regard to the studies that evaluated the filtration capacity of the resins, their penetrance, or their ability to inhibit the progression of the carious lesion, they used a common methodology, separating study groups where the use, application and result of the materials used were assessed [9,10]. 

Regarding infiltrated resins, ICON^®^ caries infiltrant (DMG, Hamburg, Germany) is the only product in the world that currently exists commercially and thus, the only resin that is studied in the set of articles reviewed. On the contrary, when combined with remineralizing agents, the use of Tooth Mousse Plus^®^ [11] or Duraphat [9] will modify the final result. It must be noted that the combination of infiltrated resins together with alternative methods of microabrasion (37% phosphoric acid or Opalustre) would be another valid option for the treatment of enamel defects. Likewise, depending on the degree of the lesion, the combination of infiltrating resins with dental whitening procedures should also be considered [7,11].

The study population, or selected sample, share some common characteristics with the studies where an experimental approach has been carried out. Usually, the treatment is recommended for young patients with young, temporary or permanent dentition with enamel defects or incipient caries lesions in upper anterior incisors [12].

Table 2 presents the results of the included studies that produced significant differences regarding the improvement in quality of life by eliminating opacities or enamel defects of the upper anterior teeth. It makes reference to the combination of the resin with the remineralizing agents and the better results such combination provides, as well as to the evaluation of the infiltration of the resins and their capacity for deepening by way of the porosities of the enamel [15].

### 3.3. Quality Assessment

The results of the quality assessment were estimated with reference to the methodology of the selected studies. The STROBE statement (Table 3) was used to assess the Moradi study [8]. The PEDro scale (Table 4) was used for Hasmun [7], Hasmun [13], Bagher [14], Swami [10], Memis [9], Turska [15] and Kabaktchieva [16]. This same scale was used to estimate the validity of two RCTs, Bhandari [11], Ammari [18] and Mattos-Silveira [12], and the SCED scale (Table 5) for the Muñoz [17] study. Most of the results obtained are of medium validity and quality, obtaining scores such as 13/22, 6/11 or 5/11.

## 4. Discussion

The downward trend in caries indices has shifted the attention of clinicians to enamel defects and specifically, in such favor today, MIH. This circumstance, together with the growing interest in aesthetics from an early age, has created the need to seek minimally invasive alternatives with effective results for the treatment of these lesions [1]. 

Enamel defects arise from a probable combination of systemic or environmental factors that could affect ameloblasts, resulting in abnormal enamel formation. As a probable multifactorial etiology, together with the aforementioned factors, a certain genetic origin has been reported, and there are studies that even suggest the presence of an autosomal recessive load in their etiology. The combination of enamel defects together with certain risk factors, whether they are plaque remains or deficient oral hygiene, as well as the breakage of dental material, means that the treatment requires early attention; at the same time, it demands strict, conservative behavior with the remaining dental tissue [19]. 

T.P. Croll had already introduced, in 1989, the use of microabrasive agents for the chemical and mechanical removal of intrinsic surface stains (50–250 microns). This system is characterized mainly by its effectiveness, safety, simplicity and low economic cost. However, in the presence of medium-depth opacities, it turns out to be not effective enough. This is the reason why alternative methods for the removal of deeper stains were thought of.

Infiltration using low-viscosity resins, a technique initially focused on the treatment of incipient carious lesions, has been a modified and commercially developed practice in Germany (Hamburg, Germany). The infiltrated resin is a system characterized by being microinvasive, allowing treatment to fill, reinforce and stabilize demineralization without sacrificing healthy tooth structure. In addition, it meets the prevention–restoration criteria, being able to mask opacities, which, until now, was not possible with minimally invasive dentistry.

It is essential to limit opacities and evaluate their density before treating them with this type of procedure since the success of the treatment will depend on it. Despite being a key point, there is no consensus on which is the most reliable diagnostic method. For Bhandari [11], the CIELAB system would be ideal; Kabaktchieva [16], however, used light-induced fluorescence, while Turska [15] opted for the classic visual diagnosis with magnifying glasses. All of them present the necessary requirements to carry out the desired clinical practice. Therefore, it could be thought that their combination would make up a complete diagnosis.

ICON™ resin (DMG, Hamburg, Germany), is based on the principle of masking lesions, producing changes in light scattering within the dental tissue. It is capable of blending opaque areas with healthy enamel. However, authors such as Hasmun [7] and Bhandari [11] state that the use of ICON™ together with remineralizing agents, microabrasive agents or whitening agents offers better results. Bhandari [11], with the application of the Tooth Mousse or Memis [8] with the Duraphat, concluded that its addition prior to the placement of the ICON™ modified the final result, questioning whether it is an economical, simple and truly effective treatment itself. Bagher [14] found no differences when using one remineralizer or another.

However, its effectiveness in stopping the progression of carious lesions is indisputable. Kabatchieva [16] and Turska [15], in the same year, have shown that there is no risk of sensitivity after its application, offering good results for the arrest and sealing of the lesion. Kabatchieva [16] also states that this material stops the progression of carious lesions on smooth, non-cavitated surfaces in both primary and permanent teeth, and improves the aesthetic result for up to 1 year after the procedure.

As Mattos-Silveira [12] reports in his study, despite being a conservative treatment, acceptability in children may vary due to the discomfort of the procedure and the long sessions spent in the clinic. In the same way, further limitations within a clinical practice are found that may complicate the treatment, especially when the approach is aimed at pediatric patients, like the use of absolute isolation or the requirement of anesthesia to avoid certain sensations in children. 

This leads to the question of whether its use can be aimed at any age range and for any patient, or if, on the contrary, a certain degree of maturity and necessary collaboration is required. This is, therefore, why Muñoz [17] concludes that despite having good results in their study, it is necessary to carry out more research to assess its efficacy in pediatric patients.

Regarding the improvement in the quality of life, Hasmun [7] and Moradi [8] carried out observational cross-sectional studies, obtaining positive results both in terms of acceptance by parents, which could be justified by their conservative nature and by the aesthetic requirements of today’s society, as well as promising results in aesthetic improvement.

The main limitations when carrying out this study have been the scarcity of articles that made reference to the use of infiltrated resins in pediatric patients, the heterogeneity of the articles found, and their low methodological quality. 

Due to the scarcity of articles regarding the topic analyzed and the lack of consensus regarding the treatment as well as the diagnosis, it was decided to collect all the available articles concerning such topics, even if they were of lower quality regarding the scale of scientific evaluation. 

The same happened with respect to the methodological variation of the different studies, and the disparity of the results. No comparable results were obtained that could be applied to the same statistical method in order to perform a meta-analysis. 

Similarly, the complexity of research in pediatric patients leads, on numerous occasions, to limiting the work as far as meta-analysis is concerned, preventing statistical analysis and highlighting the impossibility of their performance. 

To obtain better results, randomized clinical trials and standardized protocols would be necessary. Despite being a practice that is widely accepted by families and that provides good results, it must be kept in mind that it requires a correct prior diagnosis and the combination of other materials to obtain the desired results; therefore, its usefulness in children must be considered with caution.

## 5. Conclusions

Treatments carried out to date with the ICON™ system in pediatric patients provide better results for the treatment of Hypoplastic or hypomineralized enamel defects if combined with other Opalustre-type materials or prior remineralizers.

## Data Availability

Not applicable.
